# Magnetic field sensing of 3D printed Halbach arrays

**DOI:** 10.1038/s41598-025-32086-8

**Published:** 2025-12-13

**Authors:** Aigerim Ospanova, Alisher Konysbekov, Bartosz Pruchnik, Piotr Putek, Teodor Gotszalk, Andrzej Dziedzic, Grant Ellis, Piotr Skrzypacz

**Affiliations:** 1 School of Sciences and Humanities, Nazarbayev University, 53 Kabanbay Batyr Ave., 010000 Astana, Kazakhstan; 2 Faculty of Electronics, Photonics and Microsystems, Wrocław University of Science and Technology, ul. Janiszewskiego 11/17, 50-372 Wrocław, Poland

**Keywords:** Hall-effect sensor, Halbach array, Permanent Neodymium magnet, Magnetic flux density, Magnetic field, Magnetic scalar potential, Engineering, Mathematics and computing, Physics

## Abstract

This paper investigates magnetic field amplification in Halbach arrays. A Halbach array, composed of permanent magnets, is arranged to produce a strong magnetic field on one side and a weak field on the other. This configuration has numerous scientific and engineering applications. The literature review surveys representative implementations. In this work, we propose and validate a cost-effective approach for designing and fabricating Halbach magnet arrays. Specifically, in our experiments, we employ a low-cost Hall-effect sensor to measure the Halbach array’s magnetic flux density. Hall-effect sensors are well suited for measuring magnetic fields owing to their accuracy, ease of integration, low cost, and simplicity. Thus, analytical expressions for the magnetic flux density are derived from the magnetic scalar potential using the magnetostatic approximation to Maxwell’s equations and a Fourier-series expansion. We then determine and compare the magnetic flux density through experimental measurements, numerical simulations, and analytical calculations. Numerical simulations are performed using the open-source *Python* package *Magpylib*, followed by an exponential regression analysis of both experimental and simulated data. These procedures can be implemented without resorting to costly full three-dimensional magnetostatic simulations or specialized laboratory equipment and may be suited for imperfect physical models by inclusion of experimentally-fitted adjustment proportionality factor $\xi$. Notably, the maximum relative error between the simulation and experimental results is approximately 11% for the Halbach array with large size permanent magnets.

## Introduction

The concept of a Halbach array (HA), its distinct features, design optimization, and applications have attracted the attention of numerous researchers. It has been established that Halbach arrays exhibit a unique magnetization distribution, producing a strong magnetic flux emanating from one side and a near-zero flux from the opposite side^1^. This pattern is achieved through a specific arrangement of permanent magnets with appropriately oriented magnetization and varying geometries within the array^2^. The phenomenon of amplified one-sided magnetic flux was initially discovered by Mallinson in 1973^1^ and subsequently rediscovered independently by Klaus Halbach^3,4^. While Mallinson initially described it as a “magnetic curiosity” with limited applications, Halbach, in the 1980s, recognized its potential and popularized the array for use in particle accelerators. In fact, the ability to generate a strong, unidirectional magnetic field renders Halbach arrays valuable in various applications, including maglev trains.

The design of a Halbach array varies significantly depending on its applications. Accordingly, different numbers, sizes, materials, and shapes of permanent magnets may be employed in the array. Additionally, magnets can be arranged in varied ways, with cylindrical, linear, and spherical configurations being the most common. Magnets for the array are typically fabricated from samarium–cobalt (SmCo) or neodymium–iron–boron (NdFeB). Moreover, Halbach arrays have been used for decades in vehicles, electric machines, medical imaging, and other technologies. Researchers in^5–7^ investigated the implementation of the Halbach array and its magnetic flux density in linear motors using advanced analytical and numerical methods. Particularly noteworthy are modern approaches to energy harvesting, where enhanced magnetic fields serve as a source for improved harvester efficiency ^32^. In^5^, the magnetic field and forces of a linear motor with a Halbach array were determined analytically via the magnetic scalar potential (MSP). In this analysis, the separation of variables and a Fourier-series expansion were used to solve Laplace’s and Poisson’s equations, respectively, for magnetic flux density. Analytical results were confirmed by finite element analysis (FEA) and experiments on a linear motor prototype. The study by^6^ also focused on linear electric-motor design, where the magnetic field was obtained theoretically through the superposition principle. Similarly, Laplace’s equation was derived from Maxwell’s equations for a magnetic field. Here, however, instead of a Fourier-series expansion, a Fourier transform was used to analyze a finite-length system. In^7^, permanent-magnet Halbach arrays with and without back iron were considered. Interestingly, the authors applied synthetic boundary conditions in the MSP derivation and proposed four different topologies for linear machines with Halbach arrays, validating the analytical magnetic-field distributions with FEA. In^8^, wall-climbing robots are described, whose adhesive force is determined by the magnetic flux density of the embedded Halbach array. The authors used the MSP method and a Fourier transform, similar to^6^, and verified the analytical results with ANSYS-based FEA. In^9^, an adjustable linear Halbach array composed of uniformly magnetized rods was investigated. By rotating the rods, the magnetic field could be shifted from one side of the device to the other. The rods, modeled as line dipoles, were analyzed using the MSP method^5–8^, while the field and torque of infinite line dipoles were computed numerically with Mathematica. Another important application of Halbach arrays is energy harvesting via permanent-magnet arrays^10–13^.

In^10^, the authors enhanced output power and reduced the size of vibration energy harvesters (VEHs) by exploiting the properties of planar Halbach arrays. The magnetic flux density of the Halbach array was computed using Maxwell 3D v10, simulated in ANSYS, and then measured experimentally with a gaussmeter. In^11^, an electromagnetic VEH incorporating a seven-magnet Halbach array, a coil, and a cantilever beam was presented. The researchers developed an analytical expression for the magnetic field distribution and modeled the system in ANSOFT, achieving a stronger magnetic field, a smaller harvester footprint, and thus higher output power compared to conventional designs. Similarly,^12^ investigated an electromagnetic VEH with a permanent-magnet Halbach array, a coil, and a cantilever. In this work, a dual Halbach configuration was proposed to boost output power in accordance with Faraday’s law. Simulations showed that the magnetic field on the shared side exceeded that on the non-shared sides. Likewise, in^13^, the authors proposed dual Halbach arrays, suspended on two magnetic springs. Under external vibration, the dual arrays oscillated between the springs. As a result, the concentrated flux induced in the copper coils increased the electromagnetic coupling. Furthermore, FEA modeling and prototyping were carried out for the optimized VEH.

The works^14–17^ investigated practical uses of permanent-magnet fields in electrodynamic suspension (EDS) systems. In^14^, a Halbach array surrounded by conductor coils was modeled. In particular, the static magnetic field was calculated using the Biot–Savart law and a surface-current method, while the dynamic field was analyzed through a Fourier transform. Subsequently, parameter effects on system performance were examined, and the design was shown to be suitable for high-speed systems. The paper^15^ further explored Halbach arrays above a conductive plate in EDS, proposing a hybrid permanent-magnet array with overlying coils. In this work, eddy-current forces were derived with a second-order 3D vector-potential method, and the magnetic flux density was computed using the Biot–Savart law and surface-current method, with ANSOFT-based FEA providing validation. Likewise,^16^ presented an EDS system with a permanent-magnet Halbach array for maglev trains, describing the magnetic field using Melcher’s transfer-relation theorem and validating results with high-speed rotary-type dynamic test equipment. The authors of^17^ compared an EDS system using a high-temperature superconducting (HTSC) magnet with one using a Halbach array, analyzing magnetic fields and eddy currents theoretically (via Fourier transform) and numerically (via FEA), with experimental verification through a rotary-type test wheel.

Building on these analytical foundations^1,3,4^, the present work proposes and validates a practical, low-cost, and regression-based characterization framework for Halbach arrays. Specifically, we investigate two different Halbach arrays consisting of large and medium-sized permanent magnets, employ a high-sensitivity Hall-effect sensor for experimental flux density measurements, and compare the results with open-source numerical simulations performed using Magpylib (v5.1.1). We further apply exponential regression analysis to the experimental and simulated data, thereby establishing a quantitative relationship between analytical, numerical, and experimental approaches. Unlike prior studies, our methodology does not require costly full-wave electromagnetic simulations or specialized laboratory equipment.

The novelty of this work lies in demonstrating that a simple regression-based workflow, driven directly by Hall-sensor measurements, can reliably characterize practical, finite Halbach arrays. This application-oriented framework bridges low-cost experimental data, open-source simulations, and analytical models, providing a deployable alternative to purely analytical or high-end simulation-based design. Moreover, our deterministic approach can be readily extended to a stochastic setting, allowing the impact of parameter uncertainties on Halbach-array performance to be quantified. Finally, the developed model can also serve as a coarse yet representative and reliable surrogate model for use in space-mapping multi-objective optimization frameworks with high cost computational analysis such as FEA, particularly for designs subject to manufacturing-induced geometric and material uncertainties.

In particular, the employed Hall-sensor is a transducer that detects the presence and strength of a magnetic field using the Hall effect. It converts magnetic field variations into an electrical signal (voltage or digital output) for measurement and control applications. When a current-carrying conductor is placed in a magnetic field perpendicular to the current flow, a voltage (the Hall voltage) is generated across the conductor. This voltage is proportional to the strength of the magnetic field ($\vec {B}$), the current flowing through the conductor, and the material’s Hall coefficient. In analog (linear) Hall sensors, the output voltage varies continuously with magnetic field strength, allowing precise position and current sensing. Digital (switch) Hall sensors are characterized by a high/low output signal when a threshold magnetic field is detected, and are used for proximity and speed detection (e.g., in smartphones, automotive systems). Latch-type Hall sensors remain in a latched state until an opposite-polarity magnetic field is applied, and are widely employed in brushless DC motors and encoders.

Importantly, the outcomes of our research are applicable to energy harvesting systems, electric motors and generators based on new magnetic materials, and magnetic resonance imaging (MRI) systems, but also to nanometrology ^18–21^. These results are supported by the measured data. Furthermore, the measurement and analysis techniques described here can be readily implemented without costly full-wave electromagnetic simulations or expensive laboratory equipment.

The paper is organized as follows. In Section Mathematical model of Halbach array, we present the mathematical model of the Halbach array. Section Experimental set-up describes the experimental setup, and Section Experimental and simulation results reports the experimental and simulation results. In Section Discussion of results, we perform exponential regression on the data and discuss the findings. Finally, Section Conclusions summarizes the conclusions.

## Mathematical model of Halbach array

This section outlines the working principles and theoretical considerations of the linear Halbach array. Various configurations of Halbach arrays exist, depending on the intended application. Consistent with studies previously discussed ^1,3,4^, given the magnetization vector $\vec {M}$ of a permanent magnet Halbach array, the corresponding magnetic field strength $\vec {H}$ can be expressed in terms of the scalar magnetostatic potential (MSP) $\phi$.

The magnetic flux density, magnetic field strength and magnetization terms are related as follows^1^

1 $$\begin{aligned} \vec {B}=\mu _0(\vec {H}+\vec {M})\,, \end{aligned}$$

where $\vec {B}$ denotes the magnetic flux density, $\mu _0=4\pi \times 10^{-7},\text {H/m}$ is the permeability of free space, and $\vec {H}$ represents the magnetic field strength. Furthermore, according to Maxwell’s equations in the magneto-static regime, the magnetic field strength and magnetic flux density satisfy

2 $$\begin{aligned} \nabla \times \vec {H}=\vec {0}\quad \text {and}\quad \vec {\nabla }\cdot \vec {B}=0\,, \end{aligned}$$

respectively. Thus, since $\mu _0$ is isotropic, we obtain

3 $$\begin{aligned} \vec {\nabla }\cdot \vec {H}=-\vec {\nabla }\cdot \vec {M}. \end{aligned}$$

As a result, the magnetic field strength can be expressed as the negative gradient of the MSP

4 $$\begin{aligned} \vec {H}=-\vec {\nabla }\phi \,. \end{aligned}$$

Finally, substituting Eq.(4) into Eq.(3) yields

5 $$\begin{aligned} \vec {\nabla }\cdot (\vec {\nabla }\phi )=\vec {\nabla }^2\phi =\vec {\nabla }\cdot \vec {M}\,. \end{aligned}$$

### Magnetization model and effective-wavelength calibration

In this work, we model the linear Halbach array as a two-dimensional (2D), *y*-invariant structure in the *x*–*z* plane. In this configuration, the remanent magnetization can be expressed as ^1^

6 $$\begin{aligned} \vec {M}(x) =[M_x,M_y,M_z]^T=\bigl [M_0\sin (kx),\, 0,\, -M_0\cos (kx)\bigr ]^T, \end{aligned}$$

where $M_0$ is the amplitude of the rotating magnetization, defined as $M_0 \approx \frac{|\vec {M}|}{\mu _0}$ [A/m] for magnetic materials with $\mu _r\approx 1$, see, e.g., ^1,3,4^, and $k=k_c$ is the *calibrated* wavenumber^33^. Note that the choice of the sign in this expression determines the rotation direction of the magnetization and, consequently, the side of the array that is field-free. Thus, reversing the rotation reverses the one-sidedness.

Let $\tau$ denote the pitch of the magnet pole in Halbach array and $\lambda _0=2\tau$ the theoretical spatial wavelength of the fundamental spatial harmonic, see, e.g., ^27^. We then define the basic wavelength $\lambda _0$ and its wavenumber $k_0$, as well as their physics-adjusted counterparts: calibrated wavelength $\lambda _c$ and its wavenumber $k_c$ by

7 $$\begin{aligned} k_c=\frac{2\pi }{\lambda _c},\qquad \lambda _c=\xi \,\lambda _0,\qquad \lambda _0=2\tau , \end{aligned}$$

so that

8 $$\begin{aligned} k_c=\frac{k_0}{\xi },\qquad k_0=\frac{\pi }{\tau }. \end{aligned}$$

The adjustment for physical model is provided by the calibration factor $\xi \geqslant 1$, which accounts for systematic deviations from the ideal, infinitely periodic model. These deviations include finite-length effects, since only $m = 5$ periods are considered, measurement lift-off, the 3D fixture and width fringing, magnet segmentation and tolerances, and other experimental non-idealities.

**Fig. 1:**
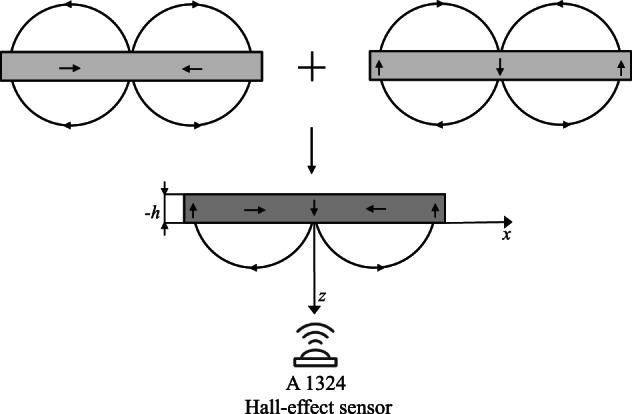
Combined magnetization pattern with one-sided field in Halbach array^1^.

In our experiment, we denote array length as $L=m\tau$ (see Fig. 1), so the ideal single harmonic of magnetic field spatial wave, which would be equal $k_0$ is in the superposition with wave lengths originating from windowing effects. Therefore, we consider the remanent magnetization phrazed as $M(x)=M_0\cos (k_0 x)\,W(x)$ on total device length $[-L/2,L/2]$ with window *W*(*x*). Furthermore, let *A*(*k*) be the Fourier transform of *M*(*x*). The remanent magnetization in the air gap at height *h* is then a superposition of spatial harmonics, each decaying as $e^{-|k|h}$. For a rectangular window $W(x)=\textbf{1}_{[-L/2,L/2]}(x)$, a single-*k* regression is well approximated by the filtered spectral centroid ^24,25^

9 $$\begin{aligned} k_{\textrm{eff}}(m,h)\approx \frac{\displaystyle \int _{0}^{\infty } k\,|A(k)|^{2}\,\textrm{e}^{-2kh}\,dk}{\displaystyle \int _{0}^{\infty } |A(k)|^{2}\,\textrm{e}^{-2kh}\,dk} \approx \frac{\displaystyle \int _{0}^{\infty } k\,\operatorname {sinc}^{2}\!\Big (\tfrac{(k-k_0)L_m}{2}\Big )\,e^{-2kh}\,dk}{\displaystyle \int _{0}^{\infty } \operatorname {sinc}^{2}\!\Big (\tfrac{(k-k_0)L_m}{2}\Big )\,e^{-2kh}\,dk}, \end{aligned}$$

where the calibration factor is defined as $\xi =\frac{k_0}{k_{\textrm{eff}}},\;\lambda _c=\xi \,\lambda _0$, since the positive-frequency lobe satisfies

$$|A(k)|^{2}\ \propto \ \operatorname {sinc}^{2}\!\Big (\tfrac{(k-k_0)L_m}{2}\Big )\,,$$

see, e.g., ^22,26^.

**Remark on the validity of the two-dimensional model.** The formulation above is 2D and assumes an infinitely long, perfectly periodic HA that is invariant along the *y*-direction. As such, it provides a leading-order description that is most accurate in the central region of the array, sufficiently far from the physical ends. In our setup with only five periods, finite-length end effects and edge fringing modify the local field, effectively broadening the spatial spectrum and slightly altering the apparent decay rate. In the present work, these deviations from the idealised model are partly absorbed into the calibrated wavelength $\lambda _c=\xi \,\lambda _0$ (through the factor $\xi$) and into the spectral-based effective wavenumber, while the remaining discrepancies are quantified and discussed in the results and discussion sections.

### Magneto-static theory for planar magnetized structure

The MSP $\phi$ and fields on both sides of permanent magnet array will be determined as solutions to the boundary value problem (BVP) for Poisson’s equation^1^. Since $\vec {M}\ne \vec {0}$, the scalar potential within the Halbach array can be expressed as^23^

10 $$\begin{aligned} \vec {\nabla }^2\phi _{HA}=M_0k\cos (kx)\,, \end{aligned}$$

where $\phi _{HA}$ is MSP within the Halbach array (HA). In contrast, since $\vec {M}=\textbf{0}$ outside the HA, the scalar potential of the strong and weak sides of the the Halbach array satisfies the BVP for Laplace’s equation

11 $$\begin{aligned} \vec {\nabla }^2\phi _{s}=0\quad \text {and}\quad \vec {\nabla }^2\phi _{w}=0\,, \end{aligned}$$

where $\phi _s$ and $\phi _w$ are MSP of strong and weak sides of the array respectively. The general solutions for the scalar potentials are then

12 $$\begin{aligned} \begin{array}{@{}l@{\;}c@{\;}l@{}} \phi _{s} & = & \left( Ae^{-kz}+Be^{kz}\right) \cos (kx)\,,\\ \phi _{HA} & = & \left( Ce^{-kz}+De^{kz}-\frac{M_o}{k}\right) \cos (kx)\,,\\ \phi _{w} & = & \left( Ee^{-kz}+Fe^{kz}\right) \cos (kx)\,, \end{array} \end{aligned}$$

where the six constants *A*-*F* are determined by the six boundary conditions.

We also impose the following condition as $|z|\rightarrow \infty$

13 $$\begin{aligned} \phi _{s}=\phi _{w}=0\quad \text {as}\quad z\rightarrow \pm \infty \,. \end{aligned}$$

Furthermore, on the surface of the Halbach array, magnetic scalar potentials become

14 $$\begin{aligned} {\begin{matrix} \phi _{s}&=\phi _{HA}\quad \text {for}\quad z=0\,,\quad \text {and}\quad \phi _{w}=\phi _{HA}\quad \text {for}\quad z=-h\,, \end{matrix}} \end{aligned}$$

where *h* is the thickness of the Halbach array. The normal flux density at $z=0$ and $z=h$ has the following form

15 $$\begin{aligned} \begin{aligned} -\dfrac{\partial }{\partial z}(\phi _s)&=-\dfrac{\partial }{\partial z}(\phi _{HA})\, {-}\, M_0\cos (kx)\,,\quad z=0\,,\\ -\dfrac{\partial }{\partial z}(\phi _w)&=-\dfrac{\partial }{\partial z}(\phi _{HA})\, {-}\, M_0\cos (kx)\,,\quad z=-h\,, \end{aligned} \end{aligned}$$

from which we infer the following solutions

16 $$\begin{aligned} \begin{array}{@{}l@{\;}c@{\;}l@{}} \phi _{s}& = & -\frac{M_0}{k}(1-e^{-kh})e^{-kz}\cos (kx)\,,\\ \phi _{HA}& = & \frac{M_0}{k}(e^{-k(h+z)}-1)\cos (kx)\,,\\ \phi _{w}& = & 0\,. \end{array} \end{aligned}$$

Physically, the combination of the magnetic fluxes produced by permanent magnets in the arrays are magnetized along the *x*- and *z*-axis and permanent magnet arrays is magnetized along the *z*-axes, with the permanent magnet array itself magnetized along the *z*-axis, which is presented in Fig. 1. The magnetic flux in -*z* direction of the magnetization $\vec {M}$ is canceled out and the magnetic flux in +*z* direction remains^23^.

Ultimately, it can be deduced from Eq. (16) that the *x*-component of the magnetic flux density is given as

17 $$\begin{aligned} B_x =\mu _0M_0(1-e^{-kh})e^{-kz}\sin {(kx)}\,. \end{aligned}$$

As a result, the magnitude of the *x*-component of the magnetic flux density along the *z*-axis at $x=x_M$ is decaying exponentially with the distance *z* as follows

18 $$\begin{aligned} |B_x|\bigr \arrowvert _{x=x_M}=\frac{\mu _0M_0}{\sqrt{2}}\,(1-e^{-kh})e^{-kz}\,, \end{aligned}$$

where we set $x_M=\frac{5}{8}\lambda _c\approx \frac{5}{4}\tau$, which corresponds to the measurement line above the central magnet of the finite five-period array; this choice fixes the phase of the sinusoidal model at the actual sensor location, where end effects are minimal and the 2D approximation is most accurate.

## Experimental set-up

**Fig. 2:**
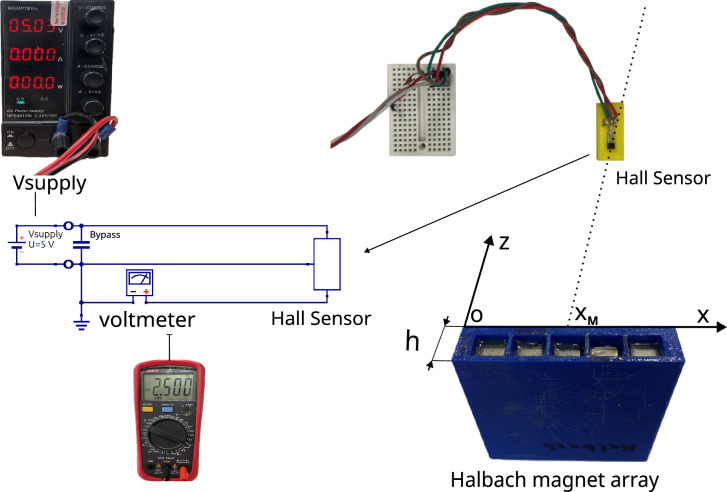
Magnetization pattern with one-sided field in Halbach array^1^.

In this section, we present the experimental results of the magnetic field measurements for the linear Halbach array, depicted in Fig. 2. In particular, the measurement setup consists of a Halbach magnet array mounted in a 3D-printed fixture, an Allegro Microsystems A1324 Hall-effect sensor, a UNI-T UT39+C multimeter, a Wanptek NPS3010W power supply, a 3D-printed measurement fixture, and a prototyping breadboard. The Hall sensor used is specified by the manufacturer to operate in ± 500 G with ± 1.5% linearity sensitivity error. Quiescent voltage drift is guaranteed to be within ± 10 G range. Voltmeter has voltage accuracy ± 0.5% + 1 mV in selected measurement range. Power supply voltage stability is within ± 0.1% + 3 mV range.

**Fig. 3:**
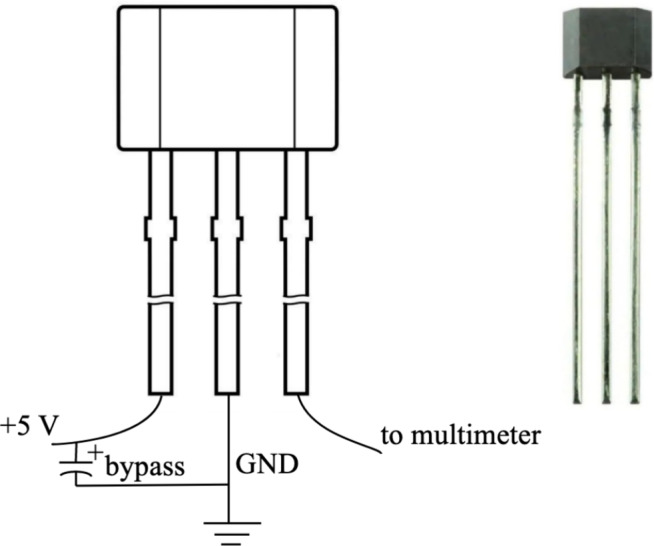
A1324 Hall-effect sensor^30^.

More precisely, two cases are considered for comparison: (i) a Halbach array arranged using five large permanent magnets, and (ii) a Halbach array arranged using five medium-sized permanent magnets. In both configurations, the magnets are oriented properly to produce a strong magnetic field on one side of the array, for example, in the $+z$ direction, while the opposite side exhibits a weak magnetic field. This effect is achieved by rotating the magnet polarity by $90^{\circ }$ between adjacent magnets. In practice, to measure the magnetic field produced on the strong side of the Halbach array, we use an Allegro Microsystems A1324 Hall-effect sensor in a 3-pin ultramini SIP through-hole package, shown in Fig. 3. As noted in^28^, Hall-effect sensors are well-suited for magnetic field measurements due to their high accuracy, ease of integration, and affordability. The output voltage of the A1324 sensor is proportional to the magnetic flux density, with the zero-field voltage equal to one-half of the supply voltage. According to the datasheet^30^, the typical sensitivity of the A1324 is 5 mV/G.

## Experimental and simulation results

This section presents the results of the measurements, simulations, and the fitting-based numerical verification. Using the Fourier transform technique to compute the magnetization $\vec {M}$ for the large finite array model discussed in Section Mathematical model of Halbach array, the window parameters are provided below in Table 1.

### Halbach array, arranged using 5 large size permanent magnets

**Table 1:** Parameters and effective-wavelength calibration for the large Halbach array ($m=5$, $\tau =\lambda /2$, $h_{\textrm{eff}}=h+\Delta g_1^{*}=16.7~\textrm{mm}$).

Window/Wavelet	$k_{\textrm{eff}}$ [m$^{-1}$]	$k_0$ [m$^{-1}$]	$\lambda _{\textrm{nominal}}$ [mm]	$\xi _{\textrm{nominal}}$ [–]
Rectangular	78.2540	123.6848	50.80	1.5813
Blackman	88.7560	123.6848	50.80	1.3936
Blackman–Harris	76.5551	123.6848	50.80	1.6159
Tukey ($\alpha =0.50$)	86.4613	123.6848	50.80	1.4301
Mexican–hat	87.7261	123.6848	50.80	1.4105

$^{*}\Delta g_1$ denotes the deviation of the sensor lift-off from its nominal value; it affects the evanescent attenuation with height and arises from manufacturing imperfections (including *casing wall thickness*/encapsulation layers, adhesive films, and fixture tolerances) and measurement uncertainties

**Fig. 4:**
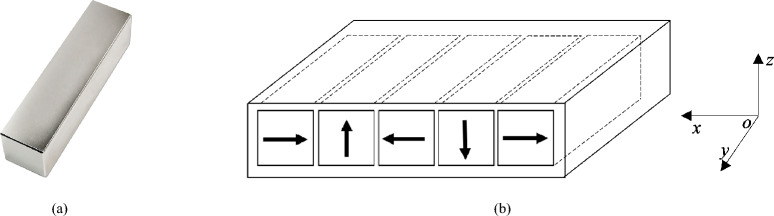
Halbach array. (**a**) Neodymium magnet. (**b**) Magnetic field orientation of each magnet.

**Fig. 5:**
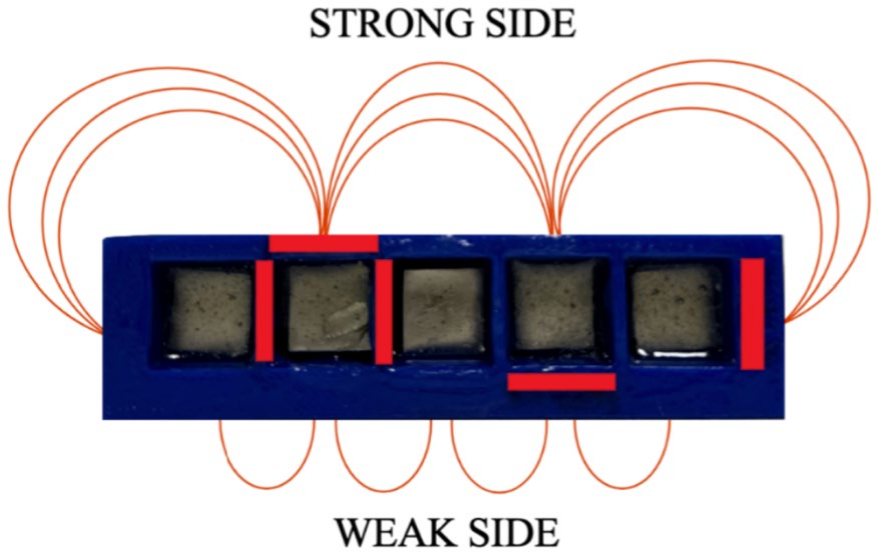
Halbach array, arranged using 5 large size permanent magnets: strong magnetic field side and weak magnetic field side.

The first Halbach array considered consists of five large cuboid permanent magnets, with the following dimensions: width $w= 12.7$ mm, height $h= 12.7$ mm, length $l = 76.2$ mm, depicted in Fig. 4a and Fig. 4b, respectively, and additionally summarized in Table 2. The magnet dimensions were chosen such that the sensor is relatively small, allowing for highly resolved field mapping. Magnets are fabricated from neodymium with nickel-copper-nickel coating and each has a magnetic flux density of 1.2 T. As stated in the previous section the permeability of free space $\mu _0=4\pi \times 10^{-7}$ H/m, while the maximum magnitude of magnetization $M_0=9.5493\cdot 10^5$ [A/m]. Moreover, the Halbach array wavelength for the five-magnet configuration is $\lambda = 50.8$ mm and the calibrated wavelength $\lambda _c = 72.649$ mm. The magnets are placed in a 3D-printed fixture with rectangular slots, with an additional 2 mm gap between them. The N52 neodymium magnets are very strong requiring caution in handling and a sturdy fixture. The fixture was fabricated using PLA filament, and the magnets were held in place with epoxy adhesive. As before, the magnets are oriented to concentrate the strong magnetic field on one side and weak magnetic field on the other, cf. in Fig. 5. We used the open source program *OpenSCAD* to design and layout the 3D printed fixtures. OpenSCAD is a programming-based CAD program for creating 3D-printing structures and CNC machining^29^. The models are fully specified in code and can be exported in common formats such as STL, DXF, or SVG. For example, an openSCAD program for a 3D printed fixture for Halbach array with five permanent magnets is shown in Appendix. In this script, a rectangular cuboid defines the outline of the fixture, and five additional cuboids define the magnet slots, which are subtracted to create openings for the magnets. Five holes are also included to allow the magnets to be removed if necessary.

**Table 2:** Characteristics of Halbach arrays.

Size of magnets	Characteristics of permanent magnets	
Large	Shape	Cuboid
	Material	Sintered NdFeB
	Coating	Ni-Cu-Ni
	Grade	N52
	Width, mm	12.7
	Height, mm	12.7
	Length, mm	76.2
	Magnetic field strength, T	1.2
Medium	Shape	Cuboid
	Material	Sintered NdFeB
	Coating	Ni-Cu-Ni
	Grade	N52
	Width, mm	10
	Height, mm	10
	Length, mm	40
	Magnetic field strength, T	1.2

As stated earlier, to measure the magnetic field generated by the Halbach array, a Hall-effect sensor is employed. A Hall-effect sensor is capable of detecting a single axial component of the magnetic flux density $\vec {B}$, depending on its location relative to the magnet. Proximity to the south pole of the magnet increases the output voltage, whereas proximity to the north pole decreases the sensor’s output voltage ^30^. Thus, the output voltage of the Hall-effect sensor, $V_H$, is proportional to the magnetic flux density *B* passing through the semiconductor material. This proportionality is defined as the magnetic sensitivity *S*. Accordingly, if the sensitivity of the sensor is known, the magnetic flux density can be determined as follows:

19 $$\begin{aligned} \begin{aligned} B=\frac{V_H-V_q}{S}\,. \end{aligned} \end{aligned}$$

By moving the sensor along the *z*-axis (cf. in Fig. 2), which is perpendicular to the strong side of the Halbach array and passes through its center, the variation of the output voltage $V_H$ from its quiescent value $V_q$ toward the supply voltage is observed. In our experiments, the quiescent value was $V_q = 2.5$ V. Thus, the $B_x$ component of the magnetic flux density $\vec {B}$ was determined for distances ranging from $z = 0.04$ m to $z = 0.10$ m. The A1324 Hall-effect sensor exhibits a sensitivity of approximately 5 mV/G, allowing measurements of magnetic flux densities up to about $\pm 500$ Gauss.

**Fig. 6:**
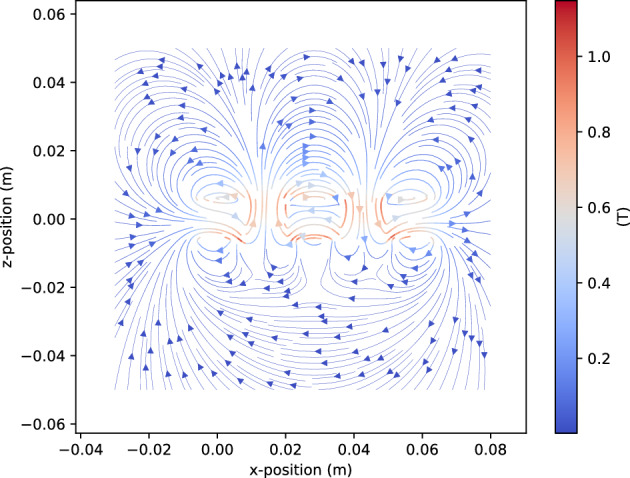
Simulated magnetic flux density of Halbach array using 5 large size permanent magnets, *Magpylib*^31^.

The corresponding simulations are conducted using the open-source Python package Magpylib, which computes magnetic fields of permanent magnets based on analytical models ^31^. The central idea behind Magpylib is to provide users with quick and convenient access to magnetic-field computation within their preferred development environment ^31^. In addition, other widely used Python tools, including NumPy and Matplotlib, are employed. The simulations replicate the experimental setup, modeling five large cuboid magnets with dimensions: width $w = 12.7$ mm, height $h = 12.7$ mm, and length $l = 76.2$ mm as presented in the Magpylib code in the Appendix. The magnets are arranged in the array with a 2 mm gap between adjacent elements. The 3D-printed PLA housing is assumed to act as free space, that is, $\mu =\mu _0$. The Halbach array is designed to concentrate a strong magnetic field on one side and a weak field on the opposite side, as described earlier (cf. in Fig. 6). The magnetic flux density $B_x$ is evaluated at the center of the Halbach array for distances ranging from $z = 0.04$ m to $z = 0.10$ m in *x*-*y*-plane, thereby allowing a 2*D* approximation to be used for the analysis. The simulation results for this configuration are then compared with the experimental measurements. The agreement is strong, with a maximum relative error of approximately 7% as summarized in Table 3.

**Table 3:** Magnetic flux density of Halbach array using 5 large size permanent magnet for various z distances.

Distance on *z*-axis, m	Output voltage $V_H$, V	Measured $B_x$, T	Magpylib $B_x$, T
0.04	0.900	0.03200	0.02984
0.05	1.829	0.01342	0.01337
0.06	2.181	0.00638	0.00636
0.07	2.341	0.00318	0.00315
0.08	2.416	0.00168	0.00161
0.09	2.463	0.00074	0.00082
0.10	2.480	0.00040	0.00041

### Halbach array, arranged using 5 medium size permanent magnets

**Table 4:** Parameters and effective-wavelength calibration for the medium Halbach array ($m=5$, $\tau =\lambda /2$, $h_{\textrm{eff}}=h+\Delta g_2^{*}=14.0~\textrm{mm}$).

Window/Wawelet	$k_{\textrm{eff}}$ [m$^{-1}$]	$k_0$ [m$^{-1}$]	$\lambda _{\textrm{nominal}}$ [mm]	$\xi _{\textrm{nominal}}$ [–]
Rectangular	91.7265	157.0796	40.00	1.7125
Blackman	109.0857	157.0796	40.00	1.4400
Blackman–Harris	91.2894	157.0796	40.00	1.7207
Mexican–hat	108.2612	157.0796	40.00	1.4509

*$\Delta g_2$ denotes the deviation of the sensor lift-off from its nominal value; it affects the evanescent attenuation with height and arises from manufacturing imperfections (including *casing wall thickness*/encapsulation layers, adhesive films, and fixture tolerances) and measurement uncertainties.

**Fig. 7:**
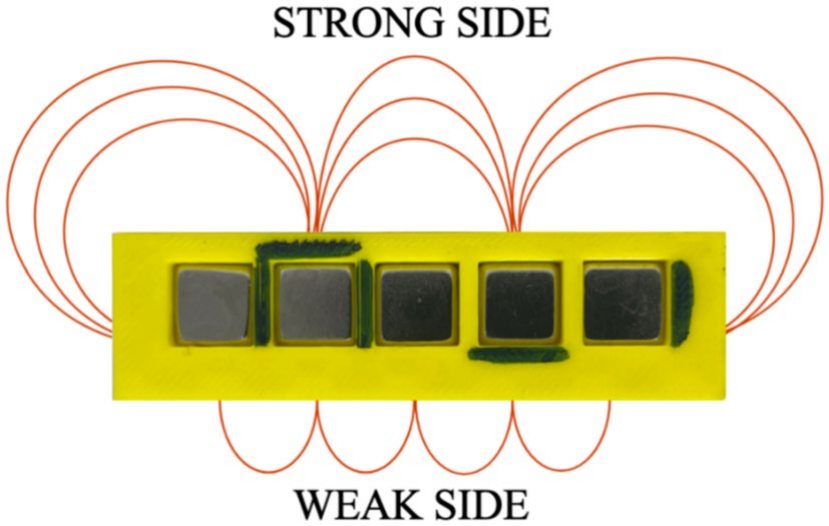
Halbach array, arranged using 5 medium size permanent magnets: strong magnetic field side and weak magnetic field side.

The considered Halbach array consists of five medium-sized N52 cuboid permanent magnets with dimensions $w = 10$ mm, $h = 10$ mm, and $l = 40$ mm, as shown in Fig. 4b and summarized in Table 2. The magnets are fabricated from neodymium with a nickel–copper–nickel coating and exhibit a magnetic field strength of approximately 1.2 T. The permeability of free space is $\mu _0 = 4\pi \times 10^{-7}$ H/m, and the maximum magnitude of magnetization is $M_0 = \frac{M}{\mu _0} = 9.5493\cdot 10^5$ [A/m]. The magnets are mounted in a 3D-printed fixture with corresponding holes and separated by a 2 mm gap. They are oriented to produce a strong magnetic field on one side of the array and a weak field on the opposite side (cf. in Fig. 7). For this configuration, the wavelength of the Halbach array arranged with five medium-sized magnets is $\lambda = 40$ mm, and the calibrated wavelength is $\lambda _c = 68.50$ mm. The window parameters used for the medium finite array model are provided below in Table 4.

**Fig. 8:**
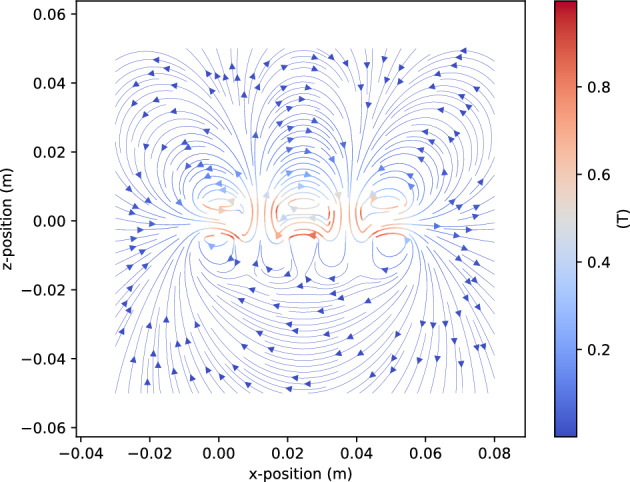
Simulated magnetic flux density of Halbach array using 5 medium size permanent magnets, *Magpylib*^31^.

As in the first experiment, an A1324 Hall-effect sensor is used to measure the magnetic field of the Halbach array. By moving the sensor along the *z*-axis, which is perpendicular to the strong side of the array and passes through its center, the variation of the output voltage $V_H$ from its quiescent value $V_q$ toward the supply voltage is observed. In this setup, the quiescent value was $V_q = 2.5$ V. From these measurements, the $B_x$ component of the magnetic flux density $\vec {B}$ is determined for distances ranging from $z = 0.04$ m to $z = 0.10$ m using Eq. (19). The A1324 Hall-effect sensor is highly sensitive, which may result in unstable readings at larger distances from the magnets. The Magpylib package is subsequently employed to perform simulations of the same configuration and compare the results with experimental data. In the simulations, five medium-sized cuboid magnets with dimensions $w = 10$ mm, $h = 10$ mm, and $l = 40$ mm are placed in the array with a 2 mm gap between them. As in the experiments, the simulated Halbach array is designed to concentrate a strong magnetic field on one side and a weak field on the other, as shown in Fig. 8.

The magnetic flux density $B_x$ is again evaluated at the center of the Halbach array for distances ranging from $z = 0.04$ m to $z = 0.10$ m. The simulation results for this configuration are then compared with the experimental measurements, as presented in Table 5.

**Table 5:** Magnetic flux density of Halbach array using 5 medium size permanent magnets for various *z* distances.

Distance on *z*-axis, m	Output voltage $V_H$, V	Measured $B_x$, T	Magpylib $B_x$, T
0.04	1.938	0.01124	0.01136
0.05	2.277	0.00446	0.00452
0.06	2.409	0.00182	0.00194
0.07	2.462	0.00076	0.00087
0.08	2.482	0.00036	0.00039
0.09	2.492	0.00016	0.00017
0.10	2.498	0.00004	0.00006

**Fig. 9:**
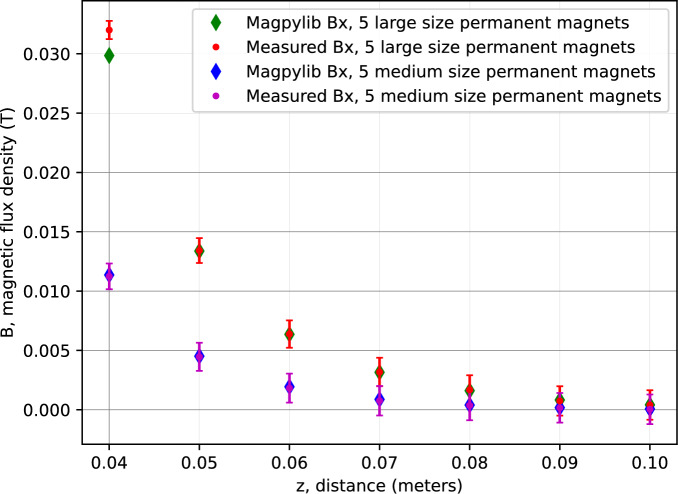
Measured magnetic flux density and *Magpylib* simulated magnetic flux density of Halbach arrays using 5 large or medium size permanent magnets.

In the end, the simulation results for both configurations, such as five large permanent magnets and five medium-sized permanent magnets, are compared with the corresponding experimental measurements, as shown in Fig. 9.

## Discussion of results

In this section, we discuss and verify the results obtained previously by fitting regression curves. Using the magnetic flux density values of the Halbach arrays from both experiments and simulations, and applying Eq. (18), we performed exponential regression for both cases:

20 $$\begin{aligned} B_x{(z)}=\alpha \,e^{-\beta z}\,, \end{aligned}$$

where $\alpha > 0$ and $\beta > 0$ are fitting parameters that depend on the magnet geometry, relative magnetic permeability, and material remanence. It is worth noting that while $\alpha$ is a convoluted constant, $\beta$ directly corresponds to the parameter *k* from Eq. (18). For the Halbach array consisting of five large permanent magnets, the fitting constants obtained from the simulations are $\alpha = 0.641$, $\beta = 76.788$, with an exponential fit accuracy of $R^2 = 0.9995$. The corresponding experimental results yield $\alpha = 0.837$, $\beta =81.734$ and $R^2 = 0.9988$. he theoretical values, calculated from Eq. (18) with an adjusted calibration coefficient for the wavelength $\xi = 1.4301$, are $\alpha = 0.914$ and $\beta = 72.649$. Thus, for the Halbach array consisting of five large permanent magnets, the theoretical expression for the magnetic flux density is obtained as

21 $$\begin{aligned} B_x{(z)} = {0.914}\, e^{-72.649\cdot z}. \end{aligned}$$

For the Halbach array consisting of five medium-sized permanent magnets, the fitting constants obtained from the simulations are $\alpha = 0.406$, $\beta = 89.451$, with an exponential fit accuracy of $R^2 = 0.9997$. The corresponding experimental results yield $\alpha = 0.432$, $\beta = 91.261$, and $R^2 = 0.9999$. The theoretical constants, derived from Eq. (18) with an adjusted calibration coefficient for the wavelength $\xi = 1.7125$, are $\alpha = 0.485$ and $\beta = 91.726$. Consequently, the theoretical expression for the magnetic flux density in this case is given by

22 $$\begin{aligned} B_x{(z)} = {0.485}\, e^{-91.726\cdot z}. \end{aligned}$$

**Fig. 10:**
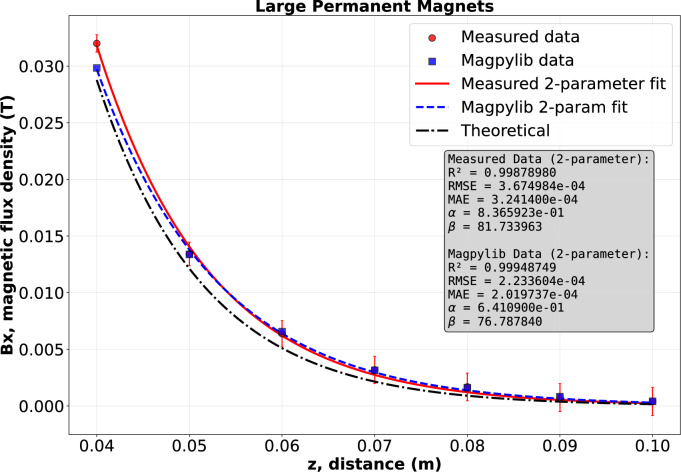
Magnetic flux density curves of Halbach array using 5 large size permanent magnet, designed based on experiment, simulation, exponential regression, and theoretical result by Eq. (21).

**Fig. 11:**
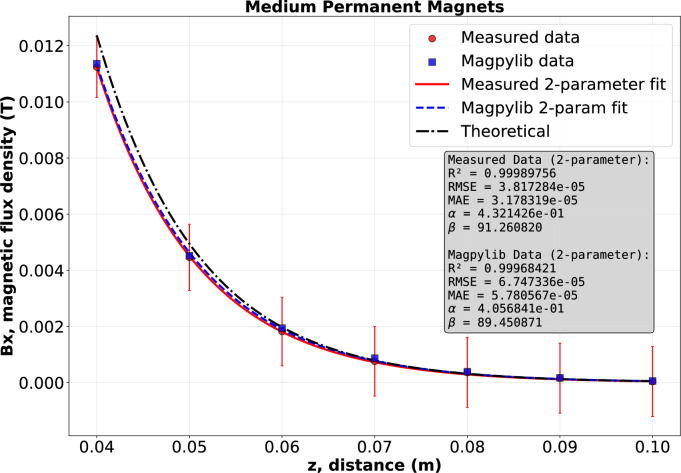
Magnetic flux density curves of Halbach array using 5 medium size permanent magnet, designed based on experiment, simulation, exponential regression, and theoretical result by Eq. (22).

It is evident that the exponential fitting function performs exceptionally well in both cases, as illustrated in Figs. 10 and 11, respectively.

**Table 6:** Parameters for $|B_x|\big |_{x=x_M}=\mu _0 M_0 \tfrac{\sqrt{2}}{2}\,(1-e^{-k h})\,e^{-k z}$ with $x_M=\tfrac{5}{8}\lambda _c$, and regression model $|B_x|=\alpha e^{-\beta z}$.

$\mu _0=4\pi \times 10^{-7}$ H/m	Measurement		Simulation		Theory	
$M_0=9.5493\cdot 10^5$ [A/m]	$\alpha$	$\beta$	$\alpha$	$\beta$	$\alpha$	$\beta$
**Large magnets** $\lambda =50.8$ mm $h=12.7$ mm $\xi =1.4301$ [–] $h_\textrm{eff}=16.70$ mm	0.837	81.734	0.641	76.788	0.914	72.649
**Medium magnets** $\lambda =40$ mm $h=10$ mm $\xi =1.7125$ [–] $h_\textrm{eff}=14$ mm	0.432	91.261	0.406	89.451	0.485	91.726

Additionally, for clarity, the fitting parameters from measurements, simulations, and the theoretical model, together with the corresponding $R^2$ values, are summarized in Table 6. The fitted coefficient $\alpha$ shows an error of about 8-11% while the decay rate $\beta$ deviates less. This behaviour is consistent with the different physical roles of the parameters in $B_x(z)=\alpha e^{-\beta z}$. The decay rate $\beta$ is mainly controlled by the geometry and the effective spatial spectrum of the array, and is therefore relatively robust, whereas the amplitude $\alpha$ is more sensitive to global scale effects such as remanent magnetization, sensor calibration, lift-off and fabrication tolerances. Consequently, $\alpha$ exhibits the larger (8–11%) discrepancy, while $\beta$ remains closer to its theoretical value.

## Conclusions

In this work, we investigated the magnetic field sensing of Halbach arrays and compared the results with analytical models of their magnetic fields using several approaches. Two Halbach arrays with different permanent-magnet sizes were analyzed experimentally with a high-sensitivity Hall-effect sensor. Following the theoretical analysis of magnetic flux density, both experimental and simulation results were presented and compared.

The supplied analysis enables viable prediction of local magnetic flux, offering a valuable tool for the precise engineering of small-scale devices, such as magMEMS. Our experiment differs from previous studies in this area by employing a Hall-effect sensor for direct magnetic field measurements. This approach minimizes the sensing footprint while allowing more accurate validation of field distribution–an aspect crucial for the design of magMEMS devices.

It was observed that the geometry of the array, as well as the magnet polarity, plays an essential role in shaping the magnetic field. The magnetic flux density was shown to increase significantly in proximity to the array. For the Halbach arrays with 5 large size and 5 medium size permanent magnets, respectively, the numerical and regression analyses are consistent, as evidenced by the simulation results and the presence of maximum relative errors of 11% and 14%, respectively. These discrepancies are primarily attributed to variations in magnet fabrication, alignment inaccuracies during assembly, and measurement tolerances.

The adjustment factor $\xi$ was found to fluctuate between cases. It is apparent, than not only magnet configuration, but also their geometry and size affect the number of superimposed spatial waves. In further works, it would be worth to find the optimization strategies for the magnetic flux to be not altered by the array geometry and length.

The above findings are of great importance for the careful formation of magnetic field lines, as these directly affect the performance of delicate micro-scale machinery. Accordingly, the presented mathematical framework is a necessary step toward the design and optimization of magMEMS devices. Since flux distribution varies strongly with distance from the magnet, minimizing both the shape and, more importantly, the dimensions of the magnets emerges as a key requirement. This aspect will be the subject of future studies.

Finally, sensing of full 3D magnetic fields using three-axis sensors will be explored in future work.

## Data Availability

Data supporting the findings of this study are provided within the manuscript and the supplementary information files, including the files required to reproduce the simulations. Measurement data are available upon request.

## References

[1] Mallinson, J. C. One-sided fluxes - A magnetic curiosity? *IEEE Trans. Magn.* **9**(4), 678–682 (1973).

[2] Blümler, P. & Federico, C. *CHAPTER 5: Hardware developments: Halbach magnet arrays, in Mobile NMR and MRI: Developments and Applications Royal Society of Chemistry* 133–157 (2015).

[3] Halbach, K. Design of permanent multipole magnets with oriented rare earth cobalt material Nucl. *Instrum. Methods* **169**(1), 1–10 (1980).

[4] Halbach, K. Applications of permanent magnets in accelerators and electron storage rings. *J. Appl. Phys.* **57**(1), 3605–3608 (1984).

[5] Silveira, M. A. D., Marques, L. D., Flores Filho, A. F. & Treviso, F. Development of an analytical method to predict the behaviour of the magnetic field in PM linear motors with Halbach array. *J. Microw. Optoelectron. Electromagn. Appl.* **16**(01), 132–153 (2017).

[6] Lee, M. G., Lee, S. Q. & Gweon, D. G. Analysis of Halbach magnet array and its application to linear motor. *Mechatronics* **14**(1), 115–128 (2004).

[7] Jin, P., Yuan, Y., Lin, H., Fang, S. & Ho, S. L. General analytical method for magnetic field analysis of Halbach magnet arrays based on magnetic scalar potential. *J. Magn.* **18**(2), 95–104 (2013).

[8] Jiao, S., Zhang, X., Zhang, X., Jia, J. & Zhang, M. Magnetic circuit analysis of Halbach array and improvement of permanent magnetic adsorption device for wall-climbing Robot. *Symmetry* **14**(2), 429 (2022).

[9] Hilton, J. E. & McMurry, S. M. An adjustable linear Halbach array JJ. *Magn. Magn. Mater.* **324**(13), 2051–2056 (2012).

[10] Zhu, D., Beeby, S., Tudor, J. & Harris, N. Vibration energy harvesting using the Halbach array smart mater. *Struct.* **21**(7), 075020 (2012).

[11] Qiu, J. et al. Low-frequency resonant electromagnetic vibration energy harvester employing the Halbach arrays for intelligent wireless sensor networks. *IEEE Trans. Magn.* **51**(11), 1–4 (2015).26203196

[12] Liu, X. et al. Design and optimization of an electromagnetic vibration energy harvester using dual Halbach arrays. *IEEE Trans. Magn.* **51**(11), 1–4 (2015).26203196

[13] Salauddin, M., Halim, M. A. & Park, J. Y. A. Magnetic-spring-based, low-frequency-vibration energy harvester comprising a dual Halbach array. *Smart Mater. Struct.* **25**(9), 095017 (2016).

[14] Luo, C., Zhang, K., Duan, J. & Jing, Y. Study of permanent magnet electrodynamic suspension system with a novel Halbach array. *J. Electr. Eng. Technol.* **15**, 969–977 (2020).

[15] Luo, C., Zhang, K., Zhang, W. & Jing, Y. 3D analytical model of permanent magnet and electromagnetic hybrid Halbach array electrodynamic suspension system. *J. Electr. Eng. Technol.* **15**, 1713–1721 (2020).

[16] Cho, H. W., Han, H. S., Bang, J. S., Sung, H. K. & Kim, B. H. Characteristicanalysis of electrodynamic suspension device with permanent magnet Halbach array. *J. Appl. Phys.* **105**(7), 92–95 (2009).

[17] Cho, H. W., Bae, D. K., Sung, H. K. & Lee, J. Experimental Study on the Electrodynamic Suspension System with HTSC and PM Halbach Array Magnets. *IEEE Trans. Appl. Supercond.* **18**(2), 808–811 (2008).

[18] Orłowska, K. et al. A method of magnetic field measurement in a scanning electron microscope using a microcantilever magnetometer. *Metrol. Meas. Syst.* **27**(1), 141–149 (2020).

[19] Zhao, L., He, J.-H., Skrzypacz, P. S., Kuangaliyeva, D., Ellis, G., Pruchnik, B. & Putek, P. “Optimizing Dynamic Pull-in Threshold and Periodic Trajectories for Magnetically Actuated MEMS (magMEMS) in Wearable Sensors,” *Front. Phys.*, **13**, 1638299 (2025).

[20] Skrzypacz, P., Ellis, G., Pruchnik, B. & Putek, P. Generalized analysis of dynamic pull-in for singular magMEMS and MEMS oscillators. *Sci. Rep.*, **15**(1), 23691 (2025). 10.1038/s41598-025-09515-9 PMC12222746 40604216

[21] Skrzypacz, P. S., Putek, P. A., Pruchnik, B. Cz., Turganov, A., Ellis, G. A. & Gotszalk, T. P. Analysis of dynamic pull-in for lumped MEMS model of atomic force microscope with constant magnetic excitation. *J. Sound Vib.* **617**, 119215 (2025).

[22] Abramowitz, M. & Stegun, I. A. *Handbook of Mathematical Functions with Formulas, Graphs, and Mathematical Tables* Vol. 55 (Government Printing Office, U.S, 1948).

[23] Shute, H. A., Mallinson, J. C., Wilton, D. T. & Mapps, D. J. One-sided fluxes in planar, cylindrical, and spherical magnetized structures. *IEEE Trans. Magn.* **36**(2), 440–451 (2002).

[24] Jackson, J. D. *Classical Electrodynamics* 3rd edn. (Wiley, New York, NY, USA, 1998).

[25] Harris, F. J. On the use of windows for harmonic analysis with the discrete fourier transform. *Proc. IEEE* **66**(1), 51–83. 10.1109/PROC.1978.10837 (1978).

[26] Pruchnik, B. C., Putek, P. A. & Gotszalk, T. P. Wavelet-based information theory in quantitative assessment of AFM images’ quality. *Sci. Rep.*, **14**(1), 3996 (2024). 10.1038/s41598-024-53846-y PMC10874965 38369551

[27] Mansson, D. On the optimization of Halbach arrays as energy storage media. *PIER B* **62**, 277–288 (2015).

[28] Ramsden, E. Hall-effect Sensors: Theory and Application *Elsevier* (2011).

[29] Horvath, J. *Mastering 3D printing* Apress (2014)

[30] https://www.allegromicro.com/en/search?q=A1324

[31] Ortner, M. & Bandeira, L. G. C. *Magpylib: A Free Python Package for Magnetic Field Computation* SoftwareX 11 p.100466 (2020)

[32] Maamer, B., Tounsi, F., Kaziz, S., Jaziri, N. & Boughamoura, A. A Halbach cylinder-based system for energy harvesting from rotational motion with high power density. *Elsevier Sensors and Actuators A: Physical* **337**, 2022 (2022).

[33] Kimel, I. & Elias, L. R. Micro-undulator fields. *Nuclear Instruments and Methods Physics Research Section A: Accelerators, Spectrometers, Detectors and Associated Equipment* **296**(1–3), 611–618 (1990).

